# Determinants of human papillomavirus vaccine hesitancy among Lebanese parents

**DOI:** 10.1371/journal.pone.0295644

**Published:** 2023-12-13

**Authors:** Ramia Zakhour, Hani Tamim, Farah Faytrouni, Maha Makki, Rayan Hojeij, Lama Charafeddine

**Affiliations:** 1 Division of Pediatric Infectious Diseases, McGovern Medical School Houston, University of Texas, Austin, Texas, United States of America; 2 Department of Internal Medicine, Clinical Research Institute, American University of Beirut Medical Center, Beirut, Lebanon; 3 College of Medicine, Alfaisal University, Riyadh, Saudi Arabia; 4 Department of Pediatrics, Medcare Medical Centers, Dr Sulaiman Al Habib Medical Group, United Arab Emirates; 5 Clinical Research Institute, American University of Beirut Medical Center, Beirut, Lebanon; National Research Centre, EGYPT

## Abstract

**Introduction:**

Human papillomavirus (HPV) is the most common sexually transmitted infection. HPV is responsible for cancer of cervix uteri. Despite its safety and immunogenicity, HPV vaccine hesitancy is one of the most challenging topics that pediatricians face.

**Methods:**

We aimed to describe the impact of knowledge, attitude, and practice towards vaccines in general, on practice related to HPV vaccination in Lebanon. A questionnaire addressed to parents of students (3–18 years of age) was distributed in 2 public and 2 private schools randomly selected from the greater Beirut area during the school year 2017–2018. Questionnaires covered knowledge, attitude, and practices of vaccination in general and HPV vaccine in particular.

**Results:**

Out of 400 distributed questionnaires, 306 (76.5%) were returned. Of the 185 parents aware of HPV vaccine, 60% hadn’t given or were not planning to give the HPV vaccine to their children. Of parents not in favor of HPV vaccine, 7.5 thought that vaccines aren’t necessary versus none among those in favor of HPV vaccine(p = 0.02). Thirteen percent of those not in favor of HPV vaccine thought that vaccines are not safe versus 2.7% in the group in favor (p = 0.02). An effect of gender on vaccine acceptance was noted: mothers vs fathers and daughters vs sons. Lack of recommendation by pediatricians and the thought that too little is known about the vaccine were the most selected reasons for parents not wanting to vaccinate their children against HPV, whereas cost and religious and cultural beliefs seemed to have no impact.

**Conclusion:**

Most parents in our study did not vaccinate or weren’t willing to vaccinate their children against HPV even when they were in favor of vaccines in general. Physician recommendation was shown to be one of the most important predictors of vaccination. Effort should be put into educating parents about the importance of the vaccine and its well-established safety and efficacy regardless of gender. Lebanese physicians should also be educated and empowered to recommend HPV vaccine more strongly and consistently.

## Introduction

Human papillomavirus (HPV), the most common sexually transmitted infection worldwide, is responsible for virtually all cases of cancer of the cervix uteri, over 90% of cases of anal cancer and a large percentage of vaginal, vulvar, penile, and oropharyngeal cancers as per the National Cancer Institute at the National Institute of Health (NIH) [1]. HPV vaccines have been licensed and widely studied since 2006 [2–4]. There is currently solid evidence supporting HPV vaccines’ safety, immunogenicity, and efficacy in reducing the circulation of vaccine preventable strains and hence the development of precancerous lesions secondary to oncogenic HPV strains [4]. HPV vaccine is currently recommended by the advisory committee on immunization practices (ACIP) and by the world health organization (WHO) for both females and males [5]. Despite these recommendations, rates of HPV vaccine coverage remain lower than any other adolescent vaccine with a global HPV immunization coverage for 2018 estimated at 12.2% only [6]. Based on the most recent UNICEF report, globally only one in eight girls are vaccinated against HPV [7]. The rates in developed countries are slightly higher ranging between 19% in France and 40–50% in the United States [8, 9].

In a meta-analysis Dorji et al reported a pooled estimate of 61.69% HPV vaccine uptake in low- and middle-income countries with a wide gap ranging between 4.72% and 87.98% [10]. Regionally the rate of immunization coverage against HPV varies between countries from less than 1% in Morocco [11] to 9.2% among female university students in Kuwait [12] and 11% in UAE [13]. As for Lebanon the latest published studies report a low vaccination rate ranging between 16 and 18.9% among 454 surveyed Lebanese female university students between 17 and 30 years who reported having received a minimum of one dose of the HPV vaccine [14, 15].

Hesitancy to HPV vaccine and to vaccination in general is present worldwide and originates from multiple levels. Identified causes for deficient HPV vaccination include lack of healthcare provider recommendation, safety concerns, parental perception of low risk of exposure to HPV, parental concerns about vaccine encouraging promiscuity, fear of needles and cost [16–19]. Less is known about the cultural influence on vaccine uptake in countries like Lebanon where cervical cancer ranks as the 10th most frequent cancer among women across all age groups and the 8th most frequent cancer among women between 15 and 44 years of age [20].

Approved HPV vaccines are available in Lebanon although not mandatory by the ministry of public health (MOPH) [21]. A study published in 2015 investigating knowledge and attitudes of Lebanese female college students towards HPV vaccination showed that only 16.5% were vaccinated against HPV [22]. A later school survey of mothers of adolescent girls conducted in a group of religious conservative schools showed that only 2.5% had given the HPV vaccine to their daughters [23]. Overall, the Lebanese population, including parents and physicians, seem to have a suboptimal knowledge of the vaccine [22–24].

There is paucity of information on knowledge and attitudes of parents in general (of both males and females and across age groups) towards HPV vaccine in Lebanon, and no study has looked at the impact of parental attitudes towards vaccination in general on acceptance of specific vaccines, like HPV vaccine. Understanding better the awareness of Lebanese parents towards HPV vaccine and recognizing the presence of specific cultural barriers can help develop targeted strategies to increase HPV vaccine uptake in Lebanon and the region. These strategies could potentially be adopted by other low-to-middle income countries or other countries from the MENA (Middle East and North Africa) region that share similar socio-cultural characteristics with Lebanon.

This study is part of a larger project investigating knowledge, attitude, and practice of Lebanese parents towards immunization in general with a focus on selected vaccines including HPV vaccine. This part of the study investigated the practice of Lebanese parents towards HPV vaccine. It also explored possible determinants of knowledge, attitude, and practice towards vaccines in general on practice related to HPV vaccination.

## Materials and methods

### Study area and design

This study was a cross—sectional survey conducted between April and November 2017. A school-based questionnaire was distributed and collected after being filled by parents across four different schools in Greater Beirut area: two private (Lycee Francais AbdelKader and Armenian Evangelical Central high school) and two public (Fakhreddine high school for girls and Raml al-Zarif secondary school). These schools are in an urban setting within the capital of the country and include students from diverse religious backgrounds.

The study team composed of bilingual doctors devised the questionnaire based on previously published questionnaires used in similar studies [25–27]. The questionnaire was developed in English and translated to Arabic. The questionnaire comprised 50 questions which addressed: knowledge, attitude and practice of parents regarding childhood vaccination in general and socio-demographic data (age, education, occupation and family income) (S1 Appendix in S1 File). Results presented in this paper focused on data collected in relation to HPV vaccine and its relation to data related to childhood vaccination in general.

### Study population and eligibility

Target population was parents of children between the ages of 3 and 18 enrolled in schools. Non-Lebanese parents and parents who were younger than 18 years of age were excluded from the study.

### Sample size determination and sampling techniques

A convenient target sample size in the original study was set at 400 based on prior similar studies [28, 29]. Post-hoc sample size calculation yielded the following: with a proportion of 60% outcome, will have a margin of error of 0.04 at 95% confidence for the 306 who were eligible for the HPV questions.

The schools were randomly selected by a computer-generated random list using a list of schools located in Greater Beirut area provided by Ministry of education. Suboptimal immunization rates and barriers to immunization are a major public health issue. Understanding parental perception of childhood immunizations and factors that encourage or discourage them from administering vaccines to their children is at the basis of improving vaccination rates in a population. The questionnaire was first piloted for face validity with 15 parents recruited from different socioeconomic levels, asking about its clarity, comprehension, length, and cultural acceptability. No modifications were required after piloting. The piloted questionnaires were not included in the final analysis but ensured that study participants were able to voice any concerns regarding the questionnaire. The questionnaire was also reviewed, and contents were approved by the responsible official in the Lebanese Ministry of Education and the Directors of the schools involved.

### Ethical considerations

The study was approved by the institutional board review at the American university of Beirut in addition to the MoPH, the ministry of education and administrations of the selected schools. Waiver of "written or verbal" informed consent was obtained from the IRB; by responding to the questionnaire individual participants gave their informed consent to include their information in the study. Questionnaires were filled out anonymously and returned in sealed envelopes to protect the privacy and confidentiality of the participants.

### Data collection

Questionnaires were self-administered and anonymous: they were distributed to students in the enrolled schools in sealed envelopes in addition to a letter instructing parents to fill out the questionnaire and return it in a sealed envelope. Filled questionnaires were then collected and returned to the study team.

Questions related to knowledge, beliefs, attitude, and trust were answered on a 5-point Likert-scale (Strongly Agree to strongly disagree). Knowledge and attitude questions were grouped into themes. For knowledge 3 themes were identified: efficacy (4 questions), safety (11 questions) and general knowledge (6 questions). For attitude, 3 themes were included: reasons (3 questions), trust (6 questions) and hesitancy (5 questions).

Answers to each question were converted into a score over 100. Basically, questions with positive answers were given a full score of 100 for “strongly agree”, 75 for “agree”, 50 for “undecided”, 25 for “disagree” and 0 for “strongly disagree”, the inverse was done for questions with negative answers. Each theme was given a final score that was equal to the average score of the individual questions listed under that theme.

### Data analysis

The IBM-Statistical Package for Social Sciences SPSS version 24 was used for data management and analysis. We calculated means and standard deviations for continuous variables and proportions for categorical variables. Associations between acceptance of Human papilloma virus and other variables were tested using Chi-squares test to compare with categorical variables whereas Student’s t-test was used to compare with continuous variables. Multivariate analysis was carried out to assess the factors associated with the outcomes. More specifically, multiple logistic regression was carried out for the practice of HPV, where results were presented as odds ratio (OR) and 95% confidence interval (CI). On the other hand, multiple linear regression was carried out for the parental knowledge and attitude towards HPB vaccine, and results are reported as beta coefficient (B) and 95% CI. A p-value of less than 0.05 was considered statistically significant.

## Results

### Participant characteristics

The overall study response rate was 76.5% [306 out of 400, 134 (43.8%) from public schools and 172 (56.2%) from private schools], however only 60.5% [185 out of 306, 68 (36.8%) from public schools and 117 (63.2%) from private schools] answered questions related to HPV vaccine and were included in the analysis (S1 Table in S1 File). Of 287 who answered the HPV questions 185 were aware of HPV vaccine (64.4%); of those 111 parents (60%) answered that they had not given or were not planning to give the HPV vaccine to their children, whereas the remaining 40% had a favorable attitude towards the vaccine.

Table 1 summarizes the sociodemographic characteristics of the participants. There was a significant difference in gender distribution between students whose parents were against HPV vaccine and those who were in favor of HPV vaccine. Parents of girls were more likely to vaccinate their child (77% compared to 60%), while parents of boys were more likely to refuse vaccinating their child (23% vs 39%).

**Table 1:** Demographic characteristics of study participants in relation to HPV vaccine acceptance.

	HPV–Vaccine	HPV+ Vaccine	p-value
	(N = 111) No. (%)	(N = 74) No. (%)	
**Gender**			
**Female**	67 (60.9)	57 (77.0)	0.02
**Child Age, years**	12.04 ± 3.18	11.22 ± 3.38	0.10
**School Grade**			0.19
Preschool	8 (7.5)	7 (9.5)	
**Elementary School**	28 (26.4)	29 (39.2)	
Middle School	60 (56.6)	30 (40.5)	
Secondary School	10 (9.4)	8 (10.8)	
**Parent filling the questionnaire, n (%)**			0.46
Father	11 (10.3)	10 (13.9)	
Mother	96 (89.7)	62 (86.1)	
**Mother’s Age, years, n (%)**			0.23
≤ 30	2 (1.8)	2 (2.7)	
>30–50	101 (91.8)	71 (95.9)	
>50	7 (6.4)	1 (1.4)	
**Mother’s employment**,			0.18
Employed	44 (40.4)	36 (48.6)	
Self-Employed	8 (7.3)	9 (12.2)	
Not Employed	57 (52.3)	29 (39.2)	
	HPV–Vaccine	HPV+ Vaccine	p-value
**Mother’s Education, n (%)**			0.66
No Formal Schooling	2 (1.8)	2 (2.7)	
High School Graduate or less	43 (39.4)	22 (30.1)	
Post-Secondary Technical	6 (5.5)	7 (9.6)	
University/College	58 (53.2)	42 (57.5)	
**Father’s Age, years, n (%)**			0.69
≤ 30	2 (1.8)	0 (0.0)	
>30–50	80 (73.4)	56 (81.2)	
>50	27 (24.8)	13 (18.8)	
**Father’s Education, n (%)**			0.11
No Formal Schooling	7 (6.4)	1 (1.4)	
High School Graduate or less	45 (41.3)	29 (42)	
Post-Secondary Technical	9 (8.3)	1 (1.4)	
University/College	48 (44.0)	38 (55.1)	
**Father’s Employment, n (%)**			0.22
Employed	70 (66.0)	38 (55.1)	
Self-Employed	35 (33.0)	29 (42.0)	
Not Employed	1 (0.9)	2 (2.9)	
**Household Income, n (%)**			**0.04**
<1000$/month	27 (33.3)	10 (17.9)	
1000–5000$/month	44 (54.3)	31 (55.4)	
>5000$/month	10 (12.3)	15 (26.8)	

HPV+ vaccine: Parents in favor of HPV vaccine HPV- vaccine: Parents not in favor of HPV vaccine.

Respondents were mostly mothers (85% compared to 60%) in the group that had not heard about the vaccine and thus could not respond to the HPV specific questions. Mean child’s age was comparable between HPV respondents and non-respondents. Overall parents who were in favor of HPV vaccine had a higher income, however within each of the public and private school groups there was no significant difference in income distribution (Table 1).

### Knowledge

**Fig 1:**
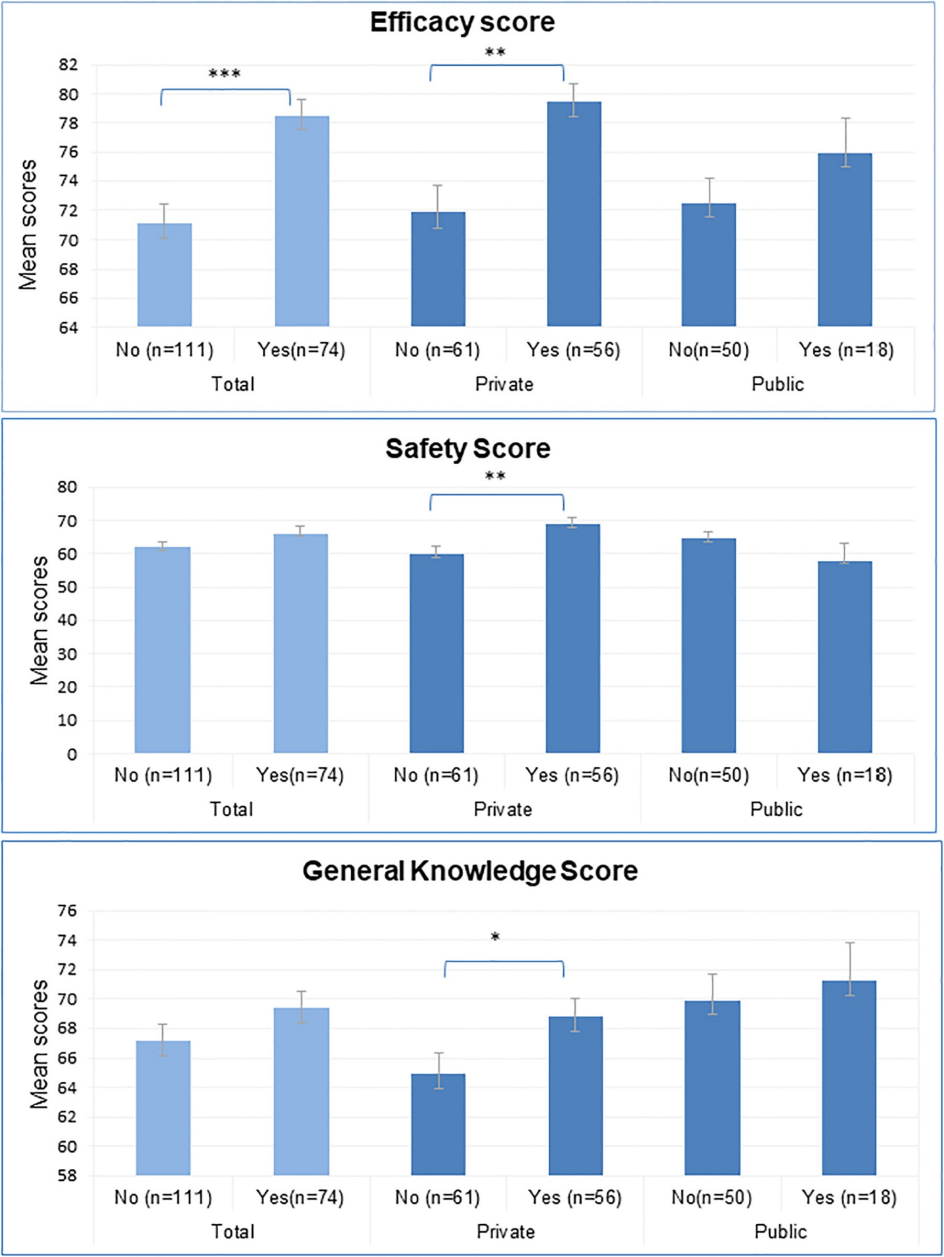
Mean knowledge scores by acceptance of HPV vaccine in private and public schools. *p<0.05; ** p<0.01; ***p<0.001.

Table 2 highlights data collected concerning parental knowledge about vaccines and acceptance of HPV vaccine. Overall scores of parents in favor of HPV vaccine reflected better general knowledge about vaccines, this was mainly driven by the private school respondents (Fig 1). Knowledge scores of parents from public school showed no significant difference between those in favor compared to those not in favor of HPV vaccine administration unlike the scores of parents from private schools (see also S2 Table in S1 File).

**Table 2:** Association between parental knowledge and HPV vaccine acceptance among Lebanese parents of school-aged children.

	HPV–Vaccine	HPV+ Vaccine	p-value
	(N = 111) No. (%)	(N = 74) No. (%)	
**Barriers n (%)**			
Lack of awareness	61 (56.5)	46 (66.7)	0.18
Financial issue	62 (57.4)	39 (56.5)	0.91
Lack access or availability of the v**accine**	21 (19.4)	22 (31.9)	0.06
No barriers	26 (24.1)	5 (7.2)	0.004
**Awareness n (%)**			
**Source of information**			
Doctor	104 (96.3)	69 (95.8)	1.00
TV	22 (20.4)	13 (17.8)	0.67
Internet	29 (26.9)	15 (20.5)	0.33
School	6 (5.6)	4 (5.5)	1.00
**Best way to raise awareness**			
Group meeting	47 (43.5)	28 (38.4)	0.49
Pamphlets	50 (46.3)	34 (47.2)	0.90
Internet	27 (25.0)	28 (38.4)	0.05
SMS	24 (22.2)	9 (12.3)	0.09
TV	46 (42.6)	35 (47.9)	0.48
Doctor	60 (55.6)	45 (61.6)	0.42
**Efficacy** *			
**Q1 Childhood vaccines are effective in protecting my child from serious disease**	87.85 ± 17.96	92.81 ± 11.39	0.02
**Q2 Having my child vaccinated is important for the health of others in my community**	79.21 ± 23.41	89.58 ± 14.38	0.001
**Safety** *			
**Q5 I don’t mind having my child receive more than 5 types of vaccine in one visit**	44.34 ± 31.68	56.51 ± 30.34	0.01
**Q6 My child is getting too many vaccines during the first two years of life which may weaken his immune system**	46.19 ± 25.89	37.33 ± 25.39	0.02
**Q8 Vaccines are not tested enough for safety**	47.75 ± 25.15	36.76 ± 26.79	0.01

HPV+ vaccine: Parents in favor of HPV vaccine HPV- vaccine: Parents not in favor of HPV vaccine;

*numbers represent mean scores ± Standard deviation.

More than 95% of participants stated getting information on vaccines from doctors with no statistically significant difference between all groups. Respondents reported the best way to raise awareness about vaccines to be through their doctor followed by television (55.6% and 42.6% respectively). Compared to parents who refused HPV vaccination, more parents in favor of the vaccine thought that childhood vaccines are effective in protecting their child and that vaccination is important for the health of others and the community. These differences were statistically significant (p = 0.02, p = 0.001 respectively).

Overall, higher scores for the safety theme were observed among those in favor of the HPV vaccine, a statistically difference was noted within the private school sample (p = 0.04) (Fig 1).

### Attitudes

**Fig 2:**
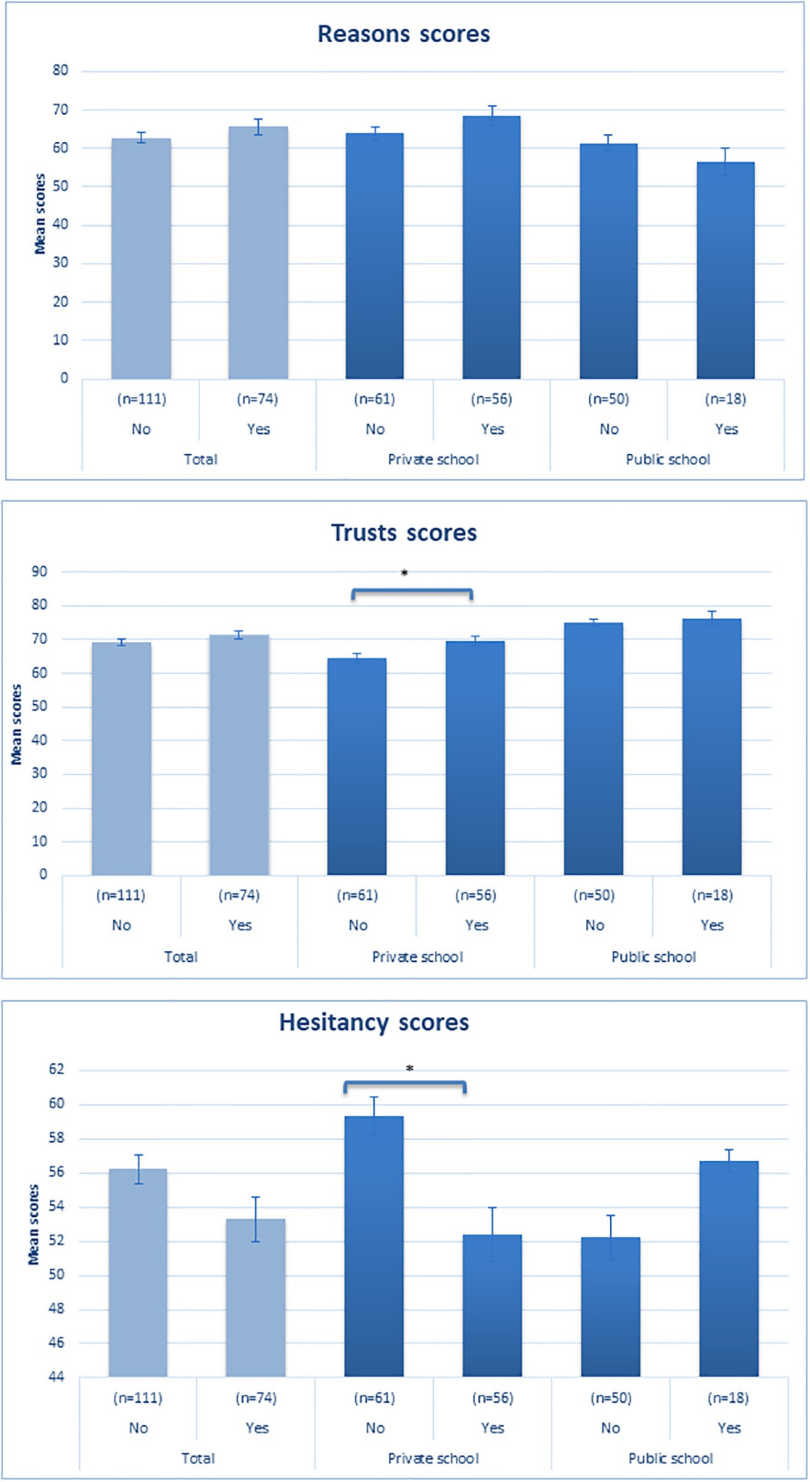
Mean attitude scores by acceptance of HPV vaccine in private and public schools. *p< 0.05.

Overall parents in favor of HPV vaccine were more likely to give recommended vaccines (p = 0.04), to follow their doctor’s recommendations (p = 0.02), and to be always in favor of vaccines (p = 0.01). They were less likely to express concerns about serious side effects of vaccines (p = 0.01) (Table 3). Of parents who were not in favor of HPV vaccine, 7.5% reported thinking that vaccines are not necessary versus none in the group in favor (p = 0.02). Of the group not in favor of HPV vaccine, 13.1% thought that vaccines are not safe versus 2.7% in the group in favor of the vaccine (p = 0.02). Fig 2 illustrates acceptance of HPV vaccine in the different groups in relation to scores related to specific aspects of parental general attitudes towards vaccines under the themes of reasons, trust, and hesitancy. Hesitancy scores were lower among people who are in favor of vaccines, and this difference was statistically significant for the private school sample(p<0.05) (Fig 2).

**Table 3:** Association between parental attitude towards vaccines and HPV vaccine acceptance among Lebanese parents of school-aged children.

	HPV–Vaccine	HPV+ Vaccine	p-value
	(N = 111) No. (%)	(N = 74) No. (%)	
**Perception of knowledge n (%)**			
Willingness to give recommended shots	86 (90.5)	66 (98.5)	0.04
Number of concomitant injections considered acceptable			0.30
1 to 2	51 (47.7)	25 (33.8)	
3 to 4	5 (4.7)	4 ()5.4	
More than 4	3 (2.8)	3 (4.1)	
Whatever the doctor recommends	48 (44.9)	42 (56.8)	
Concerns of the effects of vaccines			
Fever	94 (87.9)	62 (86.1)	0.73
Rash	31 (29.0)	19 (26.4)	0.71
Diarrhea	25 (23.4)	14 (19.4)	0.53
Inf**ection**	42 (39.3)	26 (36.1)	0.67
Too numerous	31 (28.7)	15 (20.5)	0.22
Vaccine not safe	14 (13.1)	2 (2.7)	0.02
Side effects	52 (48.6)	26 (35.6)	0.08
No concern	31 (29.0)	32 (43.8)	0.04
**Trust** *			
Q22 I trust **the information I receive about shots**	70.05 ± 20.53	72.92 ± 17.68	0.33
Q29 **Generally I do what my doctor recommends about vaccines for my child/children**	79.67 ± 19.46	86.23 ± 16.90	0.02
Q 38 **I recommend vaccination to others**	83.48 ± 25.51	89.64 ± 22.02	0.10
			
**Hesitancy** *			
Q30 **I am concerned about serious adverse effects of vaccines**.	56.37 ± 25.13	46.47 ± 25.81	0.01
Q31 **I am concerned that newer vaccines are not as safe as older vaccines because they haven’t been tested or tracked for as long**	60.28 ± 25.22	54.51 ± 26.31	0.14
Q37 **I am in favor of vaccination**.	82.26 ± 25.08	90.99 ± 19.35	0.01

HPV+ vaccine: Parents in favor of HPV vaccine HPV- vaccine: Parents not in favor of HPV vaccine;

*numbers represent mean scores ± Standard deviation.

### Practice

**Fig 3:**
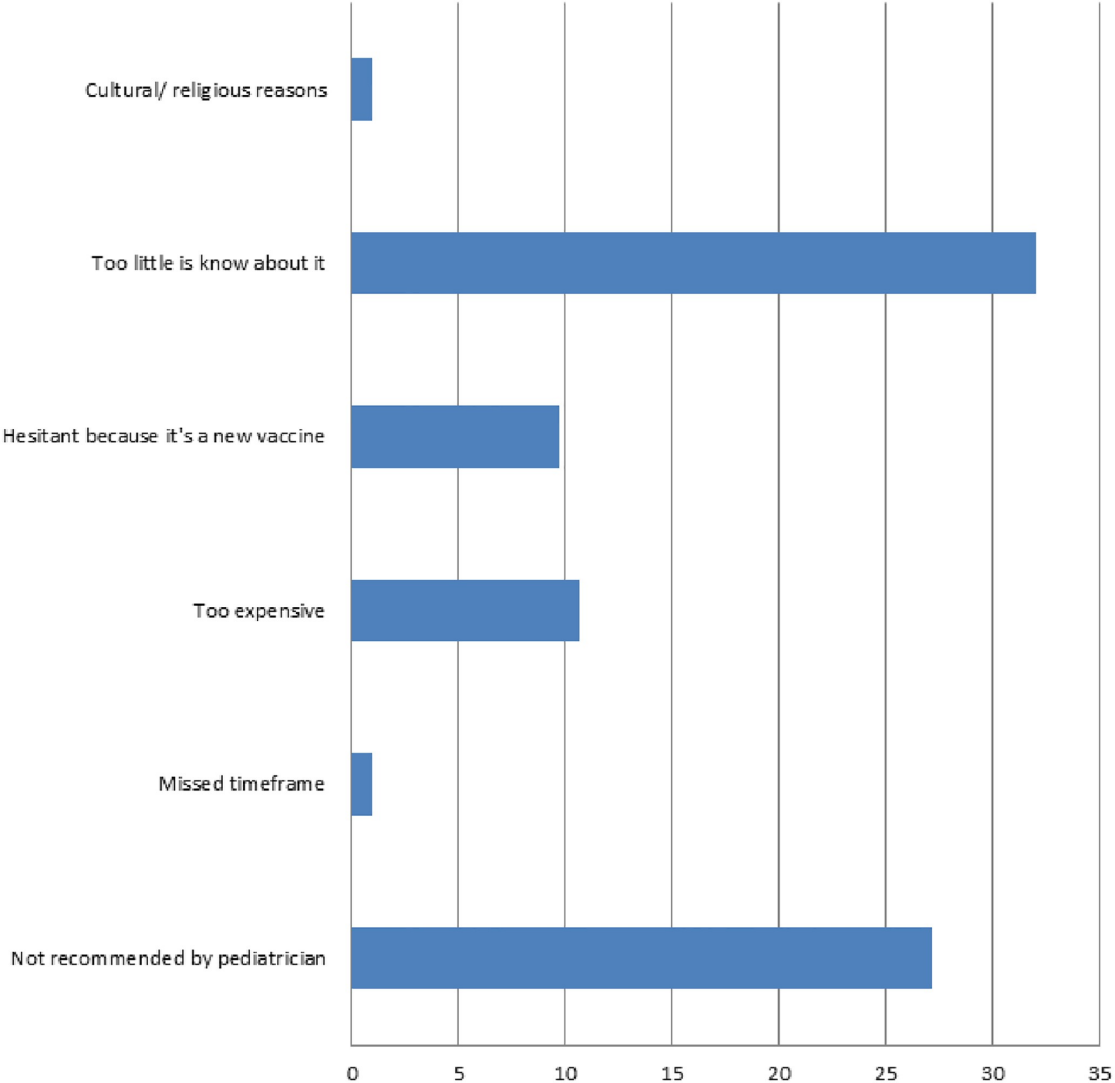
Main reasons for non-acceptance of HPV vaccine (percentage of total HPV respondents).

Parents not in favor of HPV vaccine were more likely to refuse or delay vaccines in general compared to those in favor of the vaccine (p = 0.02). Fig 3 shows reasons stated for not being in favor of HPV vaccination. Lack of recommendation by pediatrician and less awareness about the vaccine were the most selected reasons, whereas cultural/ religious reasons were rarely stated as a reason for not being in favor of the vaccine.

Tables 4 and 5 shows results of multiple regression correlating different respondent characteristics with parental knowledge, attitude, and practice towards HPV vaccine.

**Table 4:** Multiple logistic regression of factors associated with practice of HPV.

	OR (95% CI)	p
Mother’s Age	1.167 (0.062–21.867)	0.92
Mother’s employment	0.714 (0.255–2.001)	0.52
Mother’s Education	0.823 (0.286–2.369)	0.72
Father’s education	0.724 (0.243–2.154)	0.56
Parents income	1.388 (0.391–4.922)	0.61
Private vs public	4.732 (1.143–19.580)	0.032

For maternal age reference group was >30, for education reference group was university, for income reference group was >1000.

**Table 5:** Multiple linear regression of factors associated with parental knowledge and attitude towards HPV vaccine.

	Parental knowledge	p	Parental attitude	p
	B (95%CI)		B (95%CI)	
Mother’s age	-2.678 (-9.95–4.59)	0.47	-3.13 (-10.82–4.56)	0.42
Mother’s employment	3.47 (-.0.19–7.14)	0.063	0.54 (-3.30–4.39)	0.78
Mother’s Education	-0.15 (-4.39–4.09)	0.95	-1.17 (-5.63–3.29)	0.61
Father’s employment	4.27 (-8.10–16.64)	0.50	0.72 (-12.36–13.81)	0.91
Father’s education	0.90 (-2.97–4.78)	0.65	-1.34 (-5.42–2.74)	0.52
Parents income	-2.76 (-7.49–1.96)	0.25	3.51 (-1.46–8.47)	0.17
Private vs public	-5.54 (-10.62 - -0.46)	0.033	-9.10 (-14.37 - -3.83)	0.001

## Discussion

In this study, we aimed to describe the impact of knowledge, attitude, and practice towards vaccines in general on practice related to HPV vaccination among parents of school aged children in Lebanon. Most parents-respondents who were aware of HPV vaccine had not given or were not planning to give it to their children. Most parents-respondents thought that vaccines are necessary, but a small proportion said they are not safe. Lack of recommendation by pediatricians and the thought that too little is known about HPV vaccine were the most selected reasons for parents not wanting to vaccinate their children against HPV, whereas cost and religious and cultural beliefs seemed to have no impact.

A review of all studies from the MENA region looking at knowledge, awareness, and acceptability of HPV vaccine, reported overall vaccine awareness rates of 14.2–34.2% among studies focusing on parents and 32.3–63.5% among studies focusing on females only [30].

In this study 60% of responding parents knew about the HPV vaccine. This was comparable to 63.5% of Lebanese female college students from a prior study who reported being aware of the existence of the vaccine [22] but higher than the 34% awareness rate reported in a more recent publication targeting Lebanese mothers of older female school students [23]. In our study, only 40% of parents who knew about the vaccine (24% of the total participants) were willing to give or had given it to their children, a much higher rate than the 2.5% vaccine uptake reported in the above-mentioned study [23]. The differences noted may be due to the fact that the study looking at mothers’ attitudes was conducted in a group of schools with rather conservative religious socioeconomic backgrounds. A study published in 2012, from Aleppo Syria, showed that 34.2% of mothers of 6^th^ grade girls had heard about the vaccine but their level of knowledge was low [31]. A 2019 review looking at studies from Iran showed that overall population knowledge of HPV vaccine was low but attitude towards the vaccine tended to be positive; the only study from the review that looked at parental attitude towards the vaccine showed that 76% of parents had no knowledge about HPV infection [32]. In a recent study from Lebanon only 11.3% of students 15–18-year-old reported receiving at least one dose of HPV vaccine, 36.5% were not sure if they had received any doses [33].

In the previously mentioned MENA region review, out of the 18 studies reviewed only 3 looked at parental awareness and knowledge and all interviewed parents of girls only [30]. Our study showed that there remains a gender bias driving knowledge about the vaccine (mothers versus fathers) and willingness to administer the vaccine (daughters versus sons) in the Lebanese population: mothers were more likely to know about the vaccine and parents of girl students were more likely to have a favorable attitude towards the vaccine. In a review from Iran, mothers’ information score was higher than that of fathers [32]. This is also comparable to what has been reported from other areas of the world where mothers tend to be the respondents to surveys related to HPV-vaccination [34, 35] and knowledge about HPV administration to boys is significantly lower than knowledge about its administration to girls [36, 37]. Among secondary schools” Lebanese students, a low vaccination rate was noted in males (87% of vaccinated students were females and 13% were males) and 39.1% of surveyed students thought that only women can get infected. Interestingly, a recent study from Turkey surveying 18–29-year-old university students in health sciences found that only 10% thought HPV infects only women and 82% of males had heard about HPV vaccine compared to 60% of female students included [38].

Most stated reasons for non-acceptance of HPV vaccine in our study were the lack of knowledge and provider recommendation, both of which may be related as doctors are expected to educate patients about the vaccine when recommending it and since physicians were reported as an entrusted source of medical information. Only 21.9% of Lebanese secondary school students reported hearing about the HPV vaccine from their doctor, whereas internet was their primary source of knowledge (48%) [33]. This may be due to a lack of knowledge of primary care physicians about the vaccine or lack of empowerment in recommending the vaccine. A study from Turkey surveying physicians and nurses’ attitudes as parents found that 62.5% of physicians and 74.2% of nurses would not give their children HPV vaccine; 70% and 53.9% respectively would however consider it if it was included in the routine vaccination schedule [38]. A survey of Saudi pediatrician and family physicians showed that 58.38% had a good knowledge score of HPV vaccine and only 66.5% recommended the vaccine for girls 12–15 y of age, they were more likely to recommended it to older girls [39]. Our findings and findings from these studies suggest that physician education is needed to empower them to recommend and educate parents and children and adolescents about HPV vaccine. Inclusion of the vaccine in official immunization guidelines and health records even if the vaccine is not provided free of charge as part of national immunization programs may help enforce its recommendation.

Surprisingly, only 10% of parents not in favor of the vaccine stated cost and only 1% stated cultural and religious reasons being behind their decision. Currently, in Lebanon, HPV vaccine is not part of the national immunization program and is not subsidized by the government. Parents would have to pay out of pocket for the vaccine, the cost of a single dose of HPV vaccine is around 175 US dollars. Prior studies on the effect of culture and religion on HPV vaccine acceptance and uptake have shown variable results with some showing religious beliefs to increase uptake of vaccine [40], other showing the opposite [41], and lastly some studies showing no effect of religious beliefs on HPV vaccine acceptance [40].

Knowledge scores from private schools reflected a better general knowledge about vaccines to be associated with higher acceptance of HPV vaccine, this did not hold true for the public-school group where knowledge was comparable between parents in favor or not in favor of HPV vaccine. This might be due to the smaller sample size not allowing for detection of a significant difference, or to the fact that in the public-school group the decision to give HPV vaccine is driven by factors other than knowledge about vaccines in general. Similarly, some of the differences in attitudes noted between the in favor and against groups were significant for the overall group and for private schools, but not the public-school group, although for most variables trends like those seen in the private and overall group were noted.

In the public-school group not in favor of HPV vaccines, rates of parents who considered themselves always in favor of vaccines and not hesitant was high and comparable to those who were in favor of the HPV vaccine overall showing again that the public-school group had an attitude towards HPV vaccine not explained by its general attitude towards vaccination. In Lebanon families attending public schools tend to be from a less favorable socioeconomic status. Studying further decision-making drivers to HPV vaccine acceptance in that population should be undertaken. An understated role of financial barriers or religious and cultural barrier may be one possibility. However, similar to our results, a previous study exploring knowledge and attitudes of Lebanese female college students towards HPV vaccine showed that vaccine awareness was the main driver of the intent to get vaccinated rather than religion or economic status [22].

The strength of this study lies in the fact that it captured parents’ KAP about HPV vaccines in school aged children as opposed to college or university students. It also captured information from different socio-economic backgrounds and highlighted the attitude and practice of parents towards HPV vaccine for both boys and girls across age groups. Additionally, it allowed cross-examination of parental acceptance of HPV vaccine with their knowledge, attitude, and practice towards vaccines in general. An overall good knowledge and positive attitude towards vaccination leads to better acceptance of HPV vaccine.

One limitation of the study is that the target population was not met and for HPV in particular the population size was smaller since 40% of participants were not aware of the vaccine and thus could not be included in the analysis. Another limitation of the study was the geographic limitation to one urban setting area of Lebanon. Future larger studies in different areas of Lebanon and the region will give more insight into knowledge, attitudes, and practice of Lebanese parents towards childhood vaccination with a focus on HPV vaccine.

Based on our findings, efforts should be put towards raising awareness about HPV vaccine among Lebanese parents including fathers. Although mothers are usually the ones accompanying their children to doctor’s visits and making the direct decision on vaccination in our societies, decision on vaccine administration especially costly ones still is made in concertation with the father. Moreover, there is a need to improve awareness about vaccines in general and about the need to vaccinate both males and females against HPV. Across groups, parents mentioned their doctor as one of the main sources of information to acquire knowledge about vaccines. Parents in favor of HPV vaccine were more likely to follow recommendations by physician. Physicians thus play a key role in both increasing knowledge and changing parental practices. This has been shown in other studies from other parts of the world looking at factors associated with HPV vaccine uptake [18]. Educating and empowering physicians are therefore key in attaining favorable vaccine attitudes and practices.

## Conclusion

This study sheds the light on important factors that are key to future steps towards addressing HPV vaccine hesitancy in the Lebanese population and the region. The study found that lack of recommendation by physician was the main cause of non-vaccination and that there remains significant gender discrepancy in knowledge, attitude, and practice with regards to HPV vaccine. This highlights the need for efforts geared towards educating males about the vaccine and encouraging its uptake across genders as well as educating primary care physicians about the vaccine and addressing any barriers to its recommendation. These findings may help guide future efforts to improve HPV vaccination uptake in Lebanon and countries with similar cultural and socioeconomic build. Similar larger studies from other areas of Lebanon are needed to complement and confirm our findings and identify other potential barriers to HPV vaccination in Lebanon and other low-to-middle income countries.

## Supporting information

S1 File (PDF)

## Abbreviations

ACIP: Advisory Committee on Immunization Practices
HPV: Human Papillomavirus
KAP: Knowledge Attitude and Practices
LMIC: Low-Middle Income Country
SPSS: Statistical Package for Social Sciences
WHO: World Health Organization
