# Supplementary material for: Determinants of human papillomavirus vaccine hesitancy among Lebanese parents
Source: PLoS One. 2023 Dec 13;18(12):e0295644. doi: 10.1371/journal.pone.0295644 (PMC10718448; doi:10.1371/journal.pone.0295644)
Supplement: S1 File — (PDF) [file pone.0295644.s001.pdf]

Supporting information:

S1 Appendix: Questionnaire

A. Demographics

Child's information
Age : Years Months
Grade : □ Preschool □ Elementary school □ Middle school □ Secondary school
Gender : □ M □ F
Primary Nationality: □Lebanese □ non Lebanese
Child order : □ 1st □ 2nd or more
Parent's information
Parent filling the questionnaire: □ Father □ Mother
Mother age (years) □18-20 □ 20-30 □ 30-50 □ >50<br>Employment: □ Employed □ Self-employed □ Not employed What<br>is the highest degree or level of school you have completed?<br>□ No formal schooling □ Less than high school □ High school graduate □ Technical school graduate<br>□ University/college
Father age(years) □ 18-20 □ 20-30 □ 30-50 □ >50<br>Employment: □ Employed □ Self-employed □ Not employed

What is the highest degree or level of school you have completed?

□ No formal schooling □ Less than high school □ High school graduate □ Technical school graduate

□ University/college

Household income: □ <1000$/month □ 1000-5000 $/month □ >5000$/month □ No answer

B. <u>Survey Questionnaire:</u>

a. Knowledge and Believes

(Answers: strongly agree, agree, undecided, disagree, and strongly disagree)

1. Childhood vaccines are effective in protecting my child from serious disease.
2. Having my child vaccinated is important for the health of others in my community 3. It is better for my child to develop immunity by getting sick than by getting vaccinated.
4. New vaccines carry more risks than older vaccines.
5. I don’t mind having my child receive more than 5 types of vaccine in one visit (this could be one or two injections
6. My child is getting too many vaccines (10 to 15) during the first two years of life which may weaken his immune system.
7. Vaccines my cause the following: Learning disabilities, Autism, Diabetes, Sudden infant death syndrome, Other chronic diseases
8. Vaccines are not tested enough for safety.
9. Vaccines are given to children to prevent diseases that are not serious.
10. Vaccines make the immune system stronger.
11. Vaccination is for all ages, not only children.
12. No need for polio or measles vaccine anymore because of the eradication of these diseases.
13. There are situations in which you can't give live vaccines (ie: MMR,Varicella and OPV).
14. Harmful effects of vaccines are more than the benefits.

15. A healthy child does not need vaccination

16. There is a uniform immunization guideline for paediatric patients.

17. Lebanese parents are getting enough information about vaccines and their safety

18. The most common barrier(s) against vaccination in Lebanon is (are) (you can choose more than one): Lack of awareness; Financial issue; Fear; No barrier; Lack of access or availability of the vaccine

19. From where do you get the information you need about vaccines? (you can choose more than one): My Doctor; TV programs; Internet/Social Media; Friends; School; Others (Specify):

20. What do you think is the best way to raise awareness about vaccination? (you can choose more than one): Group meetings between parents and the paediatricians arranged by schools; Pamphlets from the MOH; Internet/Social Media; SMS from doctors or MOH; TV programs; Doctor offices; Others (Specify).

b. General Attitude and Trust

(Answers: strongly agree, agree, undecided, disagree, and strongly disagree)

21. The only reason I have my child get vaccinated is so that they can enter day-care or school.
22. I trust the information I receive about shots
23. There is an alternative way (hygiene or better nutrition) to protect my child from infection than vaccinating him.
24. I am satisfied with vaccination program offered by Ministry of Health.
25. I am satisfied with the way vaccines are given to my child when someone other than my pediatrician is injecting it. (i.e.: nurse, medical student, resident)
26. I have been often reluctant or hesitant to get a vaccination for my child.
27. I know parents who do not vaccinate their children because of religious or cultural reasons.
28. I am satisfied with my doctor's answers to my questions related to immunization.
29. Generally I do what my doctor recommends about vaccines for my child/children.
30. I am concerned about serious adverse effects of vaccines.
31. I am concerned that newer vaccines are not as safe as older vaccines because they haven't been tested or tracked for as long.
32. If you had another baby today, would you want him/her to get all the recommended shots?

(Yes, No, Undecided)

33. I want to know more about vaccination to help me ease my concerns. (Yes, No, Undecided)

34. When discussing the effects of vaccines with my doctor, I am concerned mostly about: (You can choose more than one) Fever; Rash; Diarrhea; Seizure; Site of injection infection; Other(Specify):

35. My greatest concern about childhood vaccines is/are: (you can choose more than one)

Too many vaccines given; Vaccines are not safe; Vaccines are not necessary; Vaccines cause diseases and long term complications; Vaccines might not prevent the disease; Vaccines cause side effects, such as fever and pain; No concerns; Other (specify)…

Answers for questions 36-40: Always, frequently, rarely and never

36. Overall, I consider myself hesitant about childhood vaccines.
37. I am in favor of vaccination.
38. I recommend vaccination to others.
39. I am concerned that childhood vaccines might not be safe. (might have a serious side effect)
40. I am concerned that a vaccine might not prevent the disease.
41. The maximum number of vaccine injections I am comfortable for my child to get per visit is: 1 to 2; 3 to 4; More than 4; Whatever the doctor recommends

c. Practice and Behavior

42. I have had to refuse or delay vaccination for my child in the past: Yes; No

43. If YES, please specify the cause (you can choose more than one answer)?

Did not think it was needed; Someone else told me that the vaccine was not safe; Did not know where to get vaccination; Heard or read negative media; Did not know where to get good; Did not think the vaccine was effective reliable information; Did not think the vaccine was safe/concerned; Fear of needles about side effects; Had a bad experience or reaction with previous vaccination; Religious reasons; Other (specify)…

44. My child has received vaccines as recommended per his/her pediatrician.

No; Yes, he/she received all; Yes, he/she received mandatory vaccines only

45. My child did receive the rotavirus vaccine: Yes; No

46. If NO, please specify the reason.

Missed the timeframe for giving it; It was too expensive at the time; Was hesitant and feared it might cause diarrhea; It was not recommended by my pediatrician; Other (specify)…

47. My child receives the flu vaccine almost every year: Yes; No .

48. If NO, please specify the reason

Missed the timeframe for giving it; It was too expensive at the time; Was hesitant and feared it might actually cause the flu; It was not recommended by my doctor; Getting the flu is not fatal; Other (specify)…

49. I vaccinated or will vaccinate my child against cervical cancer and cervical warts.

Yes; No; I am not aware of this vaccine

50. 1f No, please specify the reason

Missed the timeframe for giving it; It was too expensive at the time; Was hesitant that it is still a new vaccine and might have long term complications; It was not recommended by my doctor; Cultural/religious reasons; Too little known whether it is truly effective; Other (specify)…

S1 Table: Demographic characteristics in private and public schools in relation HPV acceptance

	Private school			Public school		
	NO (N=61) N. (%)	Yes(N=56) N. (%)	p	NO (N=50) N. (%)	YES(N=18) N. (%)	p
Gender			0.020			0.027
Male	31(51.7)	17(30.4)		12(24)	0(0.0)	
Female	29(48.3)	39(69.6)		38(76)	18(100)	
Child Age , y	10.73 ± 3.74	10.41 ± 3.46	0.64	13.56 ± 1.21	13.71 ± 1.25	0.66
School Grade			0.865			0.421
Preschool	8 (13.3)	7(12.5)		0(0.0)	0(0.0)	
Elementary School	27(45)	29(51.8)		0(0.0)	0(0.0)	
Middle School	16(26.7)	14(25)		44(95.7)	16(88.9)	
Secondary School	9(15)	6(10.7)		1(2.2)	2(11.1)	
Parent filling the questionnaire, n(%)			0.351			0.015
Father	9(15)	5(9.3)		2(4.3)	5(27.8)	
Mother	51(85)	49(90.7)		45(95.7)	13(72.2)	
Mother Age, y			0.095			1.00
18-20	0(0.0)	0(0.0)		0(0.0)	0(0.0)	

20-30	2(3.3)	2(3.6)		0(0.0)	0(0.0)	
30-50	53(88.3)	54(96.4)		48(96)	17(94.4)	
>50	5(8.3)	0(0.0)		2(4.0)	1(5.6)	
Mother’s employment,			0.424			0.073
Employed	41(69.5)	33(58.9)		3(6.0)	3(16.7)	
Self-Employed	7(11.9)	7(12.5)		1(2.0)	2(11.1)	
Not Employed	11(18.6)	16(28.6)		46(92)	13(72.2)	
Mother’s Education, n (%)			0.399			0.673
No Formal Schooling	0(0.0)	0(0.0)		2(4.1)	2(11.1)	
Less than High School	1(1.7)	3(5.5)		19(38.8)	7(38.9)	
High School Graduate	7(11.7)	8(14.5)		16(32.7)	4(22.2)	
Technical School/Graduate	4(6.7)	7(12.7)		2(4.1)	0(0.0)	
University/College	48(80)	37(67.3)		10(20.4)	18(27.8)	
Father’s Age, n(%)			0.533			0.867
18-20	0(0.0)	0(0.0)		1(2.0)	0(0.0)	

20-30	0(0.0)	0(0.0)		1(2.0)	0(0.0)	
30-50	46(78)	43(82.7)		34(68.0)	13(76.5)	
>50	13(22)	9(17.3)		14(28.0)	4(23.5)	
Father's Education, n (%)			0.207			0.146
No Formal Schooling	1(1.7)	0(0.0)		6(12.0)	1(5.9)	
Less than High School	3(5.1)	7(13.5)		25(50.0)	8(47.1)	
High School Graduate	10(16.9)	7(13.5)		7(14.0)	7(41.2)	
Technical School Graduate	3(5.1)	0(0.0)		6(12.0)	1(5.9)	
University/College	42(71.2)	38(73.1)		6(12.0)	0(0.0)	
Father's Employment, n (%)			0.090			0.317
Employed	43(75.4)	30(57.7)		27(55.1)	8(47.1)	
Self-Employed	13(22.8)	21(40.4)		22(44.9)	8(47.1)	
Not Employed	1(1.8)	1(1.9)		0(0.0)	1(5.9)	
Household Income, n (%)			0.394			1.00
<1000$/month	4(8.9)	3(6.5)		23(63.9)	7(70.0)	

1000-5000$/month	32(71.1)	28(60.9)	12(33.3)	3(30.0)
>5000$/month	9(20)	15(32.6)	1(2.8)	0(0.0)

*Mean ± Standard deviation (Range)

S2 Table : Parental knowledge and HPV vaccine acceptance in private and public schools

	Private school			Public school		
	NO (N=61)	Yes(N=56)	p	NO (N=50)	YES(N=18)	p
	N. (%)	N. (%)		N. (%)	N. (%)	
Barriers						
Lack of awareness	33(55.9)	39(72.2)	0.072	28(57.1)	7(46.7)	0.559
Financial issue	34(57.6)	32(59.3)	0.860	28(57.1)	7(46.7)	0.559
Lack access or availability of the vaccine	10(16.9)	15(27.8)	0.166	11(22.4)	7(46.7)	0.100
No barriers	16(27.1)	4(7.4)	0.006	10(20.4)	1(6.7)	0.434
Awareness						
Source of information						
Doctor	58(95.1)	53(96.4)	1.000	46(97.9)	16(94.1)	0.464
TV	10(16.4)	9(16.1)	0.962	12(25.5)	4(23.5)	1.000
Internet	19(31.1)	13(23.2)	0.336	10(21.3)	2(11.8)	0.490
School	4(6.6)	5(8.9)	0.735	9(19.1)	4(23.5)	0.732
Best way to raise awareness						
Group meeting	20(32.8)	19(33.9)	0.896	27(57.4)	9(52.9)	0.782
Pamphlets	26(42.6)	28(50.9)	0.372	24(51.1)	6(35.3)	0.396
Internet	15(24.6)	26(46.4)	0.013	12(25.5)	2(11.8)	0.319

SMS	12(19.7)	5(8.9)	0.099	12(25.5)	4(23.5)	1.000
TV	29(47.5)	29(51.8)	0.646	17(36.2)	6(35.3)	1.000
Doctor	35(57.4)	35(62.5)	0.572	25(53.2)	10(58.8)	0.780
Efficacy						
Q1 Childhood vaccines are effective in protecting my child from serious disease	85.59 ± 20.34	92.41±11.59	0.029	90.63±14.24	94.12±10.93	0.362
Q2 Having my child vaccinated is important for the health of others in my community	78.81±24.48	90.00±14.90	0.004	79.69±22.23	88.24±12.86	0.141
Safety						
Q5 I don't mind having my child receive more than 5 types of vaccine in one visit	38.98±31.91	58.48±29.10	0.001	51.06±30.38	50.00±34.23	0.905
Q6 My child is getting too many vaccines during the first two years of life which may weaken his immune system	46.61±26.03	35.26±20.10	0.010	45.65±25.98	44.11±38.05	0.879
Q8 Vaccines are not tested enough for safety	50.84±26.65	37.72±26.29	0.009	43.29±22.38	32.69±29.55	0.175

S3 Table: Association between attitude, trust and HPV vaccine in private and public schools

	Private school	Public school

	NO (N=61) N. (%)	Yes(N=56) N. (%)	p	NO (N=50) N. (%)	YES(N=18) N. (%)	p
Perception of knowledge						
Willingness to give recommended shots	45(84.9)	51(98.1)	0.031	41(97.6)	15(100)	1.000
Number of concomitant injections considered acceptable			0.102			0.579
1 to 2	35(58.3)	21(37.5)		16(34.0)	4(22.2)	
3 to 4	3(5)	3(5.4)		2(4.3)	1(5.6)	
More than 4	1(1.7)	1(1.8)		2(4.3)	2(11.1)	
Whatever the doctor recommends	21(35)	31(55.4)		27(57.4)	11(61.1)	
Concerns of the effects of vaccines						
Fever	48(80)	46(83.6)	0.614	46(97.9)	16(94.1)	0.464
Rash	16(26.7)	14(25.5)	0.882	15(31.9)	5(29.4)	1.000
Diarrhea	9(15)	11(20)	0.480	16(34.0)	3(17.6)	0.353
Infection	23(38.3)	21(38.2)	0.987	19(40.4)	5(29.4)	0.562
Too numerous	22(36.7)	12(21.4)	0.072	9(18.8)	3(17.6)	1.000
Vaccine not safe	7(11.7)	2(3.6)	0.165	7(14.9)	0(0.0)	0.175
Side effects	20(33.3)	17(30.4)	0.731	32(68.1)	9(52.9)	0.377
No concern	14(23.3)	25(44.6)	0.015	17(36.2)	7(41.2)	0.774
Trusts						

Q22 I trust the information I receive about shots	65.67±23.62	72.27±14.96	0.076	75.53±14.27	75.00±25.00	0.916
Q29 Generally I do what my doctor recommends about vaccines for my child/children	79.16±21.17	85.85±18.02	0.076	80.32±17.24	87.50±12.91	0.133
Q 38 I recommend vaccination to others	76.50 ± 28.11	86.90 ± 24.35	0.03	92.36 ± 18.50	98.15 ± 7.86	0.08
Hesitancy						
Q30 I am concerned about serious adverse effects of vaccines.	63.13±22.43	45.98±23.72	0.000	47.87±25.98	48.33±33.40	0.956
Q31 I am concerned that newer vaccines are not as safe as older vaccines because they haven't been tested or tracked for as long	67.50±22.68	51.78±25.16	0.001	51.06±25.52	64.06±28.82	0.094
Q37 I am in favor of vaccination.	75.41 ± 26.46	91.07 ± 17.40	<0.0001	90.97 ± 20.33	90.74 ± 25.06	0.97
